# Characteristics of suicide attempters with family history of suicide attempt: a retrospective chart review

**DOI:** 10.1186/1471-244X-9-32

**Published:** 2009-06-05

**Authors:** Makiko Nakagawa, Chiaki Kawanishi, Tomoki Yamada, Yoko Iwamoto, Ryoko Sato, Hana Hasegawa, Satoshi Morita, Toshinari Odawara, Yoshio Hirayasu

**Affiliations:** 1 Department of Psychiatry, Yokohama City University School of Medicine, Yokohama, Japan; 2 Psychiatric Center, Yokohama City University Medical Center, Yokohama, Japan; 3 Advanced Critical Care Medical Center, Yokohama City University Medical Center, Yokohama, Japan; 4 Department of Biostatistics and Epidemiology, Yokohama City University Medical Center, Yokohama, Japan

## Abstract

**Background:**

Family history of suicide attempt is one of the risks of suicide. We aimed at exploring the characteristics of Japanese suicide attempters with and without a family history of suicide attempt.

**Methods:**

Suicide attempters admitted to an urban emergency department from 2003 to 2008 were interviewed by two attending psychiatrists on items concerning family history of suicide attempt and other sociodemographic and clinical information. Subjects were divided into two groups based on the presence or absence of a family history of suicide attempt, and differences between the two groups were subsequently analyzed.

**Results:**

Out of the 469 suicide attempters, 70 (14.9%) had a family history of suicide attempt. A significantly higher rate of suicide motive connected with family relations (odds ratio 2.21, confidence interval 1.18–4.17, *p *< .05) as well as a significantly higher rate of deliberate self-harm (odds ratio 2.51, confidence interval 1.38–4.57, *p *< .05) were observed in patients with a family history of suicide compared to those without such history. No significant differences were observed in other items investigated.

**Conclusion:**

The present study has revealed the characteristics of suicide attempters with a family history of suicide attempt. Further understanding of the situation of such individuals is expected to lead to better treatment provision and outcomes, and family function might be a suitable focus in their treatment.

## Background

Suicide is a complicated phenomenon, and various factors are implicated in its pathogenesis [1]. Suicide risk has been reported to be associated with single marital status [2], indebtedness, unemployment [3], lower social class, male gender [4], somatic illness and psychiatric disorder [5], and history of a suicide attempt [6,7]. In addition to these risk factors, there is growing recognition that suicide and suicidal behavior (any deliberate action with potentially life-threatening consequences) tend to be familial [8-12]. Familial suicide behavior may be mediated by the transmission of endophenotypes, such as impulsivity. Environmental conditions may also result in familial transmission [13,14]. In addition, parental impulsive aggression predisposes individuals to family instability and abuse, which further increases the risk of suicidal behavior in offspring [8,15,16]. Suicidal behavior is known to aggregate in families, and both genetic and non-genetic factors responsible for familial transmission of suicidal behavior should be discernible among suicide attempters and may be suitable targets for preventive therapeutic intervention [9].

In this study, we examined the suicidal behavior and detailed sociodemographic data of suicide attempters with and without a family history of suicide attempt in order to explore our main hypothesis that suicide attempters with a family history of suicide attempt have some characteristics related to family environmental conditions. A better understanding of the situation of suicide attempters with such a history could prove useful in the provision of patient care.

## Methods

The present study was performed at the Advanced Critical Care Medical Center, Yokohama City University Medical Center, which is located in Yokohama, a mega city with a population of about 3.6 million people. The center receives all patients with potentially fatal conditions from the southern part of the city, and suicide attempters account for on average 13.0% (April 1, 2003 – March 31, 2008) of all admitted patients.

### Procedure

Between April 1, 2003 and March 31, 2008, a total of 686 suicide attempters were admitted to the center. Attempted suicide was defined as any intentional self-inflicted harm alongside suicidal ideation. Among these, 102 patients who committed suicide were excluded from the study since we could not confirm suicidal intent or obtain sufficient research information as their identities were unknown when in our care. Of the remaining 584 patients who attempted suicide, 38.2% (*n *= 223) were male and 61.8% (*n *= 361) were female, with an age ranged of 14 to 88 years and a mean of 38.0 years, standard deviation 15.9 years (*M *= 41.1, *SD *= 15.9 years for males; *M *= 36.2, *SD *= 15.5 years for females). Psychiatric diagnosis was made according to DSM-IV criteria [17] by agreement of two psychiatrists. The most common axis I diagnosis of DSM-IV was major depressive disorder (23.1%), followed by adjustment disorder (19.5%), schizophrenia (15.4%), and substance use disorder (10.4%). The most common axis II diagnosis of DSM-IV was personality disorder (32.0%), followed by mental retardation (1.2%). The breakdown of the axis II diagnosis of DSM-IV was borderline personality disorder (55%), personality disorder not otherwise specified (33%), antisocial personality disorder (9%), and others.

Patients were interviewed by two psychiatrists on the following items: 1) family history of suicide attempt, 2) living status, 3) education, 4) previous psychiatric history, 5) somatic complications, 6) method of suicide attempt, 7) history of suicide attempt, 8) history of deliberate self-harm (no suicidal ideation), and 9) motive of suicide attempt. Regarding suicide motives, patients selected the motive that corresponded most closely to their situation from the following 7 options: family relations, human relations (work place or school), male-female relationships, health issues, financial situation, work environment, or other reason.

**Figure 1:**
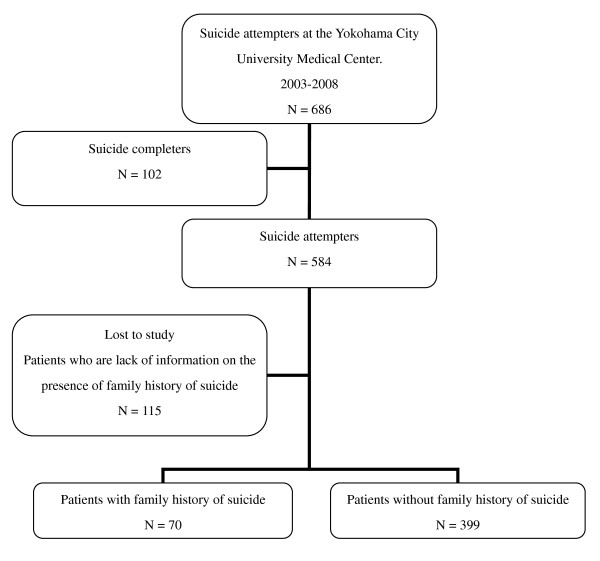
**Flow of subjects through the study**.

Subjects were divided into two groups based on the presence or absence of a family history of suicide attempt, and the differences between the two groups were subsequently analyzed. We counted every suicide attempter among a first-degree relative and grandparent. No suicides among children were reported by the patients in our sample. The flow of the patients through this study is presented in Figure 1.

### Statistical analyses

Statistical analyses were conducted using SPSS for Windows version 16.0. The chi-square test and t-test were used to compare those who reported a family history of suicide attempt and those who did not. The chi-square test was used to explore the differences between those with and without a family history of suicide in relation to gender, living status, and education. The t-test was used to compare the differences between those with and without a family history of suicide in relation to age. Further, logistic regression analysis was performed to determine differences between those with and without a family history of suicide in relation to previous psychiatry history, somatic complications, method of suicide attempt, history of suicide attempt, history of deliberate self-harm, and motive of suicide attempt. In the logistic regression model, we used age, gender, and living status as adjustment variables. A probability level of *p *< .05 was considered statistically significant.

### Ethics

The study protocol was approved by the ethics committee of Yokohama City University School of Medicine, and conforms to the provisions of the Declaration of Helsinki in 1995. We obtained informed consent from all participants and their anonymity was preserved.

## Results

Among the original sample of 584 patients, data from 115 patients (20%) were not submitted due to lack of information regarding the presence of a family history of suicide attempt. Information was lacking either because hospitalization in the emergency department was too short to obtain all information or in the case that a patient had consciousness disturbance due to head injury. Nevertheless, these untraced 115 patients did not differ significantly from the traced patients in terms of either gender or age (*p *> .05). Finally, data from 469 patients were analyzed and the results are presented below. The sample was composed of 173 (36.9%) males and 269 (63.1%) females, with an age range of 14 to 88 years and a mean of 38.1 years, standard deviation of 15.7 years (*M *= 40.6, *SD *= 15.7 years for males; *M *= 36.7, *SD *= 15.5 years for females).

**Figure 2:**
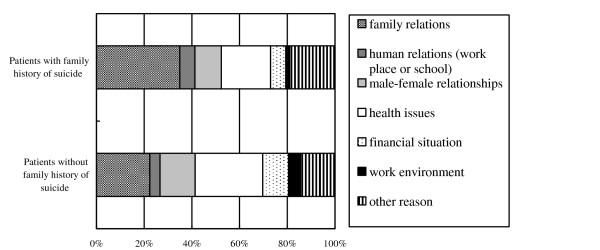
**Classified subitems of motive of suicide attempt**. The most common motive of suicide attempt concerned family relations (34.9%) in patients with a family history of suicide attempt.

Analysis revealed that 70 (14.9%) had a family history of suicide attempt and 399 (85.1%) had no such history. Sociodemographic and clinical characteristics when divided into presence or absence of a family history of suicide attempt are shown in Table 1. Figure 2 shows the breakdown of motive of suicide attempt by percentage, where the most common motive among patients with a family history of suicide attempt was revealed to be family relations (34.9%), followed by health issues (18.6%), and other reason (17.1%). For patients without a family history of suicide attempt, the most common motive of suicide attempt was health issues (28.3%), followed by family relations (22.4%), and other reason (19.0%). Thus, patients with a family history of suicide attempt showed a significantly higher rate of suicide motive connected with family relations than those without such history, with an adjusted odds ratio of 2.21 (1.18 to 4.17, *p *< .05, adjusted for age, sex, and living status), as well as a significantly higher rate of deliberate self-harm (DSH) (50% versus 34.0%, respectively), with an adjusted odds ratio of 2.51 (1.38 to 4.57, *p *< .05, adjusted for age and sex) (Table 2). Aside from these two characteristics, no significant differences between the two patient groups were observed for any other items investigated.

**Table 1:** Sociodemographic and clinical characteristics of suicide attempters, and presence/absence of family history of suicide

	Total *n *(%)	Patients with family history of suicide *n *(%)	Patients without family history of suicide *n *(%)
Living status (*n *= 453)			
Alone	100 (22.1)	14 (21.2)	86 (22.2)
Together	353 (77.9)	52 (78.8)	301 (77.8)

Education (*n *= 451)			
Compulsory education*	125 (27.7)	23 (33.8)	102 (26.6)
High school education and over	326 (72.3)	45 (66.2)	281 (73.4)

Previous psychiatric history (*n *= 467)	329 (70.4)	53 (76.8)	276 (69.3)

Somatic complications (*n *= 469)			
Permanent damage	12 (25.6)	2 (2.9)	10 (2.5)
No permanent damage			
Require in-patient treatment	45 (9.6)	4 (5.7)	41 (10.3)
Require out- patient treatment	84 (17.9)	15 (21.4)	69 (17.3)
Without physical complications	328 (69.9)	49 (70.0)	279 (69.9)

Method of suicide attempt (*n *= 469)			
Drug overdose	244 (52.0)	37 (52.9)	207 (51.9)
Laceration	71 (15.1)	12 (17.1)	59 (14.8)
Jumping from high place	58 (12.4)	9 (12.9)	49 (12.3)
Poisoning	44 (9.4)	8 (11.4)	36 (9.0)
Burning	14 (3.0)	0 (0)	14 (3.5)
Traffic death	13 (2.8)	1 (1.4)	12 (3.0)
Hanging	18 (0.2)	3 (4.3)	15 (3.8)
Drowning	4 (0.9)	0 (0)	4 (1.0)
Other	3 (0.6)	0 (0)	3 (0.8)

Previous suicide attempt (*n *= 443)	206 (44.8)	38 (55.1)	168 (43.0)

Previous deliberate self-harm (*n *= 460)	161 (36.3)	33 (50.0)	128 (34.0)

Motive of suicide attempt (*n *= 416)			
Family relations	101 (24.3)	22 (34.9)	79 (22.4)
Human relations (work place or school)	19 (4.6)	4 (6.3)	15 (4.2)
Male-female relationships	59 (14.2)	7 (11.1)	52 (14.7)
Health issues	113 (27.2)	13 (20.6)	100 (28.3)
Financial situation	42 (10.1)	4 (6.3)	38 (10.8)
Work environment	19 (4.6)	1 (1.6)	18 (5.1)
Other reason	63 (15.1)	12 (19.0)	51 (14.4)

* Compulsory education lasts for 9 years; statutory schooling ages are between 6 and 15 years in Japan.

**Table 2:** Results of examining the difference between patients with and without family history of suicide (*N *= 469)

	Adjusted OR (CI 95%)	*p *value
Deliberate self-harm^†^	2.51 (1.38–4.57)*	0.003

Motive of suicide attempt connected with family relations^‡^	2.21 (1.18–4.17)**	0.013

*Note*. * Odds ratio (OR) adjusted for sex and age.

** OR adjusted for sex, age, and living state.

† Nine of the 469 patients were excluded from the analysis due to insufficient data.

‡ Fifty-three of the 469 patients were excluded from the analysis due to insufficient data.

Confidence interval = CI.

## Discussion

This study was performed to determine whether suicide attempters with a family history of suicide attempt showed characteristics different from those without such history. Of note, this is the first study to focus on motives of suicide attempt in suicide attempters with a family history of suicide.

In this study, 14.9% of the suicide attempters at our emergency department had a family history of suicide attempt, which is similar in frequency (13.2%) to that among suicide attempters with a family history of suicide attempt recently reported by Diaconu et al [15]. The rate of suicide motive connected with family relations and the rate of the deliberate self-harm were significantly higher among patients with a family history of suicide attempt in our study. A number of studies have reported on the etiology of the familial transmission of suicidal behavior. The effects of family history are thought to be mediated through both shared biologic vulnerability and family environmental conditions [8,18-20]. Considering the factor of family environment, family function is regarded as one of the key elements [13,21]. Children and adolescents who present with deliberate self-harm often experience major life problems, especially in relationships with family members [22,23]. Family discord has consistently been shown to be both a correlate and predictor of adolescent suicidal behavior [24]. Our finding is not in conflict with these previous studies. While family dysfunction might be related to the cause of suicide, we were not aware of the details of their "family relations" motive or of whether it marked the beginnings of possible family dysfunction in each case.

Family therapy for suicide attempters and their families is beneficial for maintaining family function. Morrison et al. stated that the attempted suicide would affect the entire family, and the treatment plan for each family should be based on family interaction and the individual functioning of each member within the family [25]. Kerfoot et al. reported that family interventions are an effective means of addressing the issues associated with adolescent suicidal behavior [26]. Some of our subjects were bereaved due to family history of suicide, and in the case of bereavement, previous studies have indicated the effectiveness of intervention and social support to reduce distress and suicidal ideation [27-29]. In addition, there is also a pressing need for studies that ask those with a family history of suicide attempt themselves what has been of help or what they feel so that interventions can be designed to strengthen the natural coping efforts of families [30]. Reducing the stigma of suicidal behavior and increasing awareness of the psychological distress of individuals who experience suicidal behavior of their family will make it much easier for them to access social support. In Japan, where the increasing number of suicides is of grave concern, the National Suicide Prevention Measure Outline established in 2007 stated the need to provide care and social resources for both bereaved families and families of suicide attempters [31].

We recognize some limitations of our study. First, we did not conduct structured interviews with suicide attempters to diagnose psychiatric disorder. Hospitalization in our emergency department is too short to perform structured interviews for patients. Instead, psychiatric diagnosis was made on the consensus of two attending psychiatrists. The second limitation is that the situation of cohabitation at the time when a family member attempted suicide was unclear. The third limitation is that some of the suicide attempters may have been unaware of a family history of suicide attempt.

## Conclusion

In the emergency department, 14.9% of suicide attempters had a family history of suicide attempt. We observed significantly higher rates of suicide motive connected with family relations and of deliberate self-harm in suicide attempters with a family history of suicide attempt than in those without such history. These findings indicate that care for the suicide attempters should take into consideration a family history of suicide. Replication of these findings in future studies that perform more extensive investigation is warranted.

## Abbreviations

DSM: The Diagnostic and Statistical Manual of Mental Disorders.

## Competing interests

The authors declare that they have no competing interests.

## Authors' contributions

MN, RS, YI contributed to data collection. MN, CK, TY, HH, TO, YH wrote the analysis plan. MN and SM conducted the statistical analysis. CK discussed the ideas in paper and contributed to manuscript preparation. All authors contributed to the interpretation of the results and the final manuscripts.

## Pre-publication history

The pre-publication history for this paper can be accessed here:


